# Analysis of Clinical Characteristics, Background, and Paroxysmal Activity in EEG of Patients with Juvenile Myoclonic Epilepsy

**DOI:** 10.3390/brainsci12010029

**Published:** 2021-12-27

**Authors:** Efraín Santiago-Rodríguez, Elba Zaldívar-Uribe

**Affiliations:** Diagnóstico, Tratamiento e Investigación Neurológica, S. C. Querétaro, Santiago de Queretaro 76177, Mexico; ezu140267@gmail.com

**Keywords:** juvenile myoclonic epilepsy, paroxysmal activity, electroencephalogram, quantitative EEG analysis, polyspike and wave complexes

## Abstract

Juvenile myoclonic epilepsy (JME) appears in adolescence with myoclonic, absence, and generalized tonic clonic (GTC) seizures with paroxysmal activity of polyspike and slow wave (PSW), or spike and wave (SW) complexes in EEG. Our aim was to analyze the clinical characteristics, background EEG activity, and paroxysmal events in 41 patients with JME. Background EEG activity was analyzed with visual, quantitative (QEEG), and neurometric parameters. Our JME patients started with absence seizures at 11.4 ± 1.5 years old, myoclonic seizures at 13.6 ± 2.5 years, and GTC seizures at 15.1 ± 0.8 years. The seizures presented in awakening at 7:39 h with sleep deprivation, alcoholic beverage intake, and stress as the most frequent precipitant factors. Paroxysmal activity was of PSW and fast SW complexes with 40.5 ± 62.6 events/hour and a duration of 1.7 s. Right asymmetric paroxysmal activity was present in 68.3% of patients. Background EEG activity was abnormal in 31.7% of patients with visual analysis. With QEEG beta AP (absolute power) increase and AP delta decrease were the most frequent abnormalities found. Spectral analysis showed that 48.7% of patients had normal results, and 26.83% and 24.4% had higher and lower frequencies than 10.156 Hz, respectively. We concluded that, with visual analysis, background EEG activity was abnormal in a few patients and the abnormalities increased when QEEG was used.

## 1. Introduction

Juvenile myoclonic epilepsy (JME) is a genetically-determined disease that appears in adolescence with myoclonic seizures, absence, and generalized tonic clonic (GTC) seizures that are characteristically present for two hours after awakening. Electroencephalogram (EEG) shows paroxysmal activity of polyspike and slow wave (PSW) or fast spike and wave complexes in frontocentral leads with a frequency of three- to five-hertz and a variable duration of events [1,2,3].

JME presents during childhood or adolescence with an absence of seizures in 40% of patients, and myoclonic and GTC seizures at some point in adolescence at approximately 17 years of age [4]. Some patients with JME have a family history of disease in variable ranges, depending on the population that is studied. Currently, some genes were postulated to partially explain the origin of the disease [5].

From a physiological point of view, it has been postulated that an imbalance exists between the excitatory and inhibitory neural networks that are located in the medial frontal regions, with a strong interrelation with the thalamocortical networks that regulate the sleep–wake cycle [6,7]. This alteration has been corroborated through EEG current source analysis studies, voxel-based morphometry, magnetic resonance spectroscopy, and functional magnetic resonance [8,9,10].

Previous studies have described the clinical and EEG characteristics in different ethnic groups and countries. As expected, these characteristics present small differences between them. For example, in a population in Saudi Arabia, a higher degree of familial cases was reported with the researchers even postulating an autosomal recessive type of transmission. However, most studies agree on the type of seizure and average age at onset [11,12,13,14].

The electroencephalographic characteristics that are found in JME are consistent in two patterns: one with PSW and the other with a fast SW pattern whose sources are in the frontocentral regions. Recent studies have observed a certain degree of asymmetry and even a focal origin of paroxysmal activity modifying the classical concept of generalized seizures in JME. JME patients have been considered to have normal background EEG activity using visual analysis. Currently we have more powerful tools such as quantitative analysis (QEEG) and especially neurometric analysis that allows for the obtaining of more precise and consistent results; its usefulness has already been recognized in other brain diseases such as Alzheimer’s disease as well as attention and learning disorders. In addition, with the use of QEEG, the effects of various drugs on brain electrical activity have been more precisely evaluated, giving rise to the Quantitative Pharmaco-EEG. The analysis of background EEG activity in the JME has shown inconsistent results, in part because in adolescence the electrical activity of the brain undergoes marked changes that prevent a correct comparison of the EEG frequency bands. Neurometric analysis allows an adjustment of the normal values according to the age of the patient in such a way that the z-values that are obtained are comparable regardless of the age of the subjects that are analyzed. Therefore, the objective of this study was to describe the clinical characteristics, analyze the paroxysmal activity, and background EEG activity using visual analysis, QEEG, and especially neurometric analysis in a group of patients with JME [15,16,17,18,19,20].

## 2. Materials and Methods

The EEGs of 41 patients with a final diagnosis of JME were analyzed over a period of 10 years from 2012 to 2021 in the Clinic Neurophysiology and Neurological Private Center (Diagnóstico, Tratamiento e Investigación Neurológica). In these 10-year periods, a total of 4800 EEG studies were conducted. All patients with suspected JME underwent a clinical neurological exam by one of the authors (E.S.-R.). The patients, or their parents, signed an informed consent form, and the study was approved by the Ethical and Research Committee. The tenets of the Declaration of Helsinki were followed.

### 2.1. EEG Recordings

The EEG recordings were obtained with 19 electrodes that were placed according to the International 10–20 System using the Medicid-5 System (Neuronica Mexicana, S.A., México). Referential leads with linked ear lobes and average references were used. The gain of 19 channels was 10,000 dB; the low- and high-cut filters were 0.50 and 70 Hz, respectively; and the sample frequency was 200 Hz. The impedance of all electrodes was under 5000 Ω. The EEG recording was obtained by one of the authors (E.Z.-U.) in a quiet room in the states of rest with the eyes closed, the eyes open, hyperventilation for three minutes, one minute of hyperventilation recuperation, and photic stimulation for one minute with a stroboscopic lamp in the frequency range of 5 to 33 Hz. The total duration of the EEG recording was 20 min. On the day of the EEG recording, the patients kept their usual sleep and wake times.

### 2.2. Background EEG Activity

Background EEG activity was determined through visual and quantitative analysis (QEEG) with fast Fourier transform of 24 segments of 2.56 s in patients with their eyes closed and totally awake. The absolute power (AP), relative power (RP), and the mean frequency (MF) of the delta, theta, alpha, and beta bands were obtained. Due to the broad range of ages in our patient group, the brain oscillations had very different frequencies in normal subjects and the simple comparison of AP, RP, and MF of different bands would have produced erroneous results. Therefore, neurometric analysis was used with comparison against a normal population and the results are expressed as z-scores. The normative sample of 211 functionally healthy subjects in the age range of 5- to 97-years was obtained in random form from the universe of 116,000 inhabitants of Havana City, Cuba, with stringent criteria for inclusion where 65% of the subjects were rejected. The sample was divided into quasi-logarithmically-spaced intervals, yearly from 5 to 15.9; every two years from 16 to 19.9; every five years from 20 to 97. Age dependent quantitative norms were established for Broad Band Spectral Parameters of AP, RP, and MF and Narrow Band Spectral Parameters between 0.39–19 Hz (Medicid-5 System, Neuronica Mexicana, S.A., México). When the results were greater than 1.96 or less than −1.96, the z-scores were considered abnormal (*p* < 0.0249). In addition, with the objective of decreasing non-physiological variability and improving diagnostic precision, the subtraction of the global scale factor (GSF) was applied [21].

### 2.3. Paroxysmal Activity

A visual review of the EEGs was performed to detect paroxysmal activity of PSW or fast SW of JME by one of the authors (E.S.-R.). The number, duration, and the symmetry of paroxysmal activity were obtained. Statistical analysis included descriptive measures and the mean and standard deviation of each variable. For the analysis of the independence of the background EEG activity from antiepileptic treatment, the patients were divided into those without and with antiepileptic treatment at the time of EEG recording. The independence analysis was carried out by means of a 2 × 2 contingency table and statistical significance was determined by means of the chi-square test, taking those with *p* < 0.05 as significant.

## 3. Results

### 3.1. Clinical Characteristics

A total of 41 patients were selected, 16 men and 25 women, with a mean age of 21.1 ± 11.5 years (range of 12- to 68-years). A family history of epilepsy was present in 12 (29.2%) patients. The epilepsy age onset was of 13.6 ± 2.5 years. All the patients had myoclonic seizures with age onset at 13.6 ± 1.71 years. A total of 33 (80.4%) patients had GTC seizures with a mean onset age of 15.1 ± 0.8 years. Only nine (21.9%) patients had absence seizures with a mean age onset of 11.4 ± 1.5 years. Seizures are characteristically present in the first hours of the morning after awakening; our patients had seizures at 7:39 ± 3.2 a.m.

Patients with JME have many factors that increase the risk of seizures. In our group, the factors leading to seizures were sleep deprivation in 18 (43.9%) patients, alcoholic beverage intake in 8 (19.5%), stress in 7 (17.0%), photic stimulus in 1 (2.4%), menstruation in 1 (2.4%), and videogame playing in 1 (2.4%) patient. Only 4 patients (9.7%) had a diagnosis of JME at the time of entry into the study, and 23 (56.1%) patients had treatment with antiepileptic drugs. However, only 11 (26.8%) had treatment with valproate and 4 (9.7%) had treatment with levetiracetam, an antiepileptic drug that has demonstrated antiepileptic utility in JME. The mean daily dose of valproate was 661.7 ± 194.8 mg/day.

### 3.2. Paroxysmal Activity

**Figure 1:**
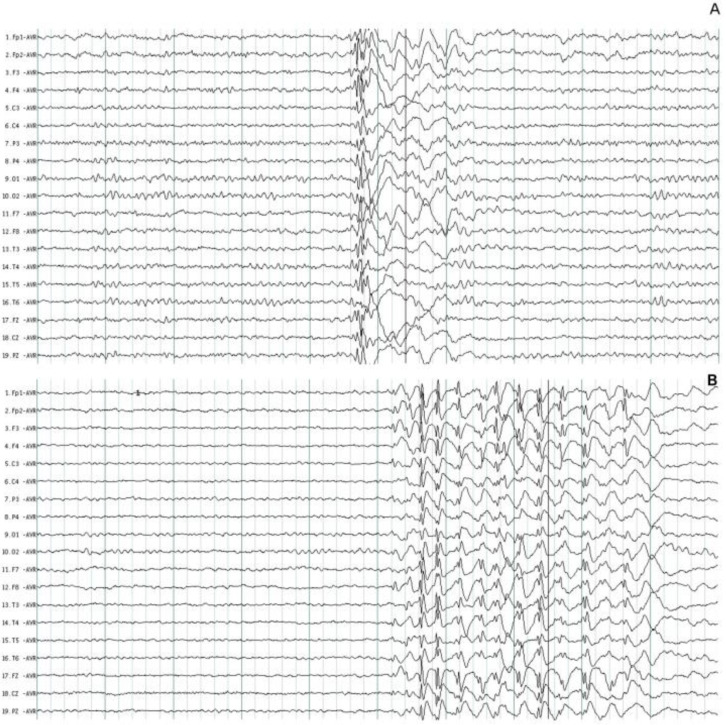
Typical paroxysmal activity in two patients with juvenile myoclonic epilepsy. (**A**) Polyspike and wave complexes of short duration. (**B**) Fast spike and wave complexes with more amplitude on the frontal leads. The normal background EEG activity was identified in two patients.

All the patients had characteristic paroxysmal activity of the PSW complex; in addition, 30 (73.1%) patients had fast SW complexes, and 22 (53.6%) had slow waves. Although the duration of the EEG recordings was between 20- and 30-min, the number of events per hour was calculated. The mean number of paroxysmal events was 40.5/h ± 62.6 with a mean duration of 1.7 ± 1.1 s, the shortest duration of events was 0.3 s, and the longest duration was 15.2 s. The accumulated duration of paroxysmal events was 20.5 s, which represents 1.7% of all EEG recordings. The paroxysmal activity had asymmetrical distribution in 35 (85.4%) patients. Asymmetrical right paroxysmal activity was shown in 28 (68.3%) patients and 7 (17.0%) on the left side. Patients with asymmetric paroxysmal activity maintained such asymmetry throughout the EEG recording, but all patients had paroxysmal events on the opposite side. Focal paroxysmal events in temporal electrodes were observed in two patients (4.8%). None of the patients had myoclonic seizures during EEG recordings. The typical paroxysmal events are shown in Figure 1.

### 3.3. Background EEG Activity

The background EEG activity was normal in 28 (68.3%) patients (Figure 1) and abnormal only in 13 (31.7%) patients during visual EEG review. In these patients, the main abnormalities were generalized theta increases and decreases in alpha activity in six (46.1%) patients; the other six (46.1%) patients had generalized beta increases, and an increase in alpha activity was observed in only one (7.8%) patient. To determine if the use of antiepileptic treatment has any effect on the baseline EEG activity, a comparison was made between the proportion of patients with and without treatment. Of the 18 patients without antiepileptic treatment, 11 had a normal EEG; the 23 patients with antiepileptic treatment had 17 patients with a normal EEG. The differences were not significant (*p* = 0.38).

**Figure 2:**
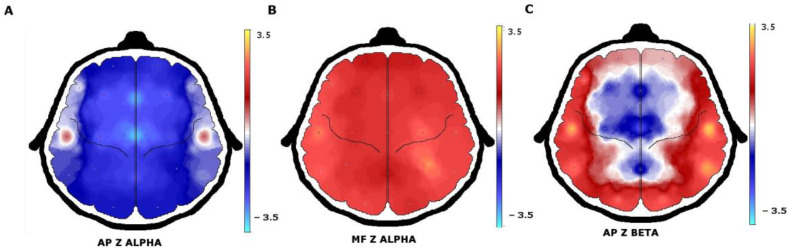
QEEG with neurometric analysis of patients with juvenile myoclonic epilepsy. (**A**) A generalized decrease in alpha absolute power in the frontocentral regions with (**B**) an increase in alpha mean frequency. (**C**) The beta absolute power increase was observed in the temporal and occipital regions.

When quantitative EEG (QEEG) and neurometric analysis were used, more EEG abnormalities were found. Comparison of the proportion of patients with normal or abnormal EEG background activity between visual EEG analysis and QEEG showed a sensitivity of 30.8% and a specificity of 50.0%. Beta AP increases and delta AP decreases were the most frequent abnormalities that were found. In a broader description, increases were observed in beta AP in 22 (53.6%) patients, theta AP in 14 (34.1%), alpha AP in 13 (31.7%), and delta AP in 6 (14.6%). In contrast, a decrease in delta AP was found in 31 (75.6%) patients, theta AP in 23 (56.0%), beta AP in 18 (43.9%), and alpha AP in 15 (36.5%) patients (Figure 2).

When the analysis was carried out in patients without and with antiepileptic treatment, normal values were observed in the delta band in four patients without treatment and with antiepileptic treatment in two patients (*p* = 0.22). In the theta band, two patients without antiepileptic treatment had normal values, and three patients with treatment had normal values (*p* = 0.85). The beta band was normal in one patient in both groups (*p* = 0.85). Only in the alpha band were the differences significant, 2 patients without antiepileptic treatment had normal values and 11 patients with treatment (*p* = 0.012).

Abnormalities in MF were much less frequent than abnormalities in AP. An increase in theta MF was observed only in 11 (26.8%) patients, alpha MF in 8 (19.5%), and beta MF and delta MF in 6 (14.6%) patients. A decrease in delta MF was found in 18 (43.9%) patients, in alpha MF in 10 (24.1%), in theta MF in 6 (14.6%), and in beta MF in 5 (12.1%) patients. The number of patients who had normal MF values of the delta (*p* = 0.73), theta (*p* = 0.087), alpha (*p* = 0.48), and beta (*p* = 0.4) bands were not significantly different in those without and with antiepileptic treatment.

**Figure 3:**
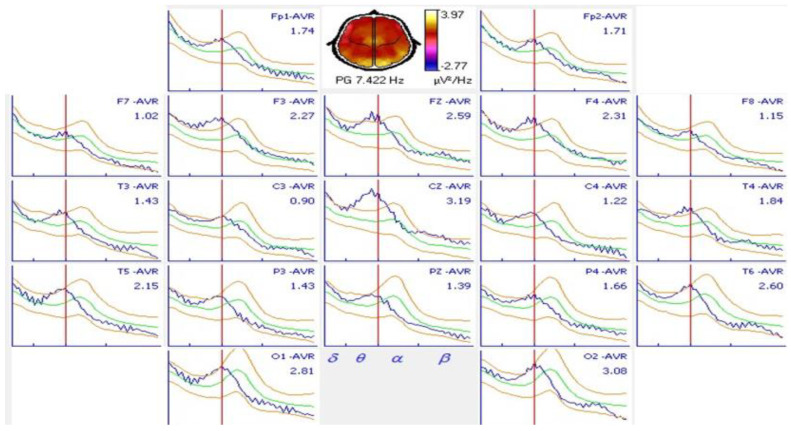
Spectral analysis of an 18-year-old patient with juvenile myoclonic epilepsy and comparison with normal values. A shift in the alpha spectral peak to a 7.422 Hz frequency was observed in all derivations. A normal alpha spectral peak at this age occurs at 10.156 Hz.

Spectral analysis showed an alpha peak in 9.91 ± 0.91 Hz with greater amplitude in the occipital regions on the O1 and O2 electrodes. Almost half of the patients (48.7%) had normal results when they were compared with the spectral alpha peak in 9.766 or 10.156 Hz. Only 11 (26.8%) patients had shifts at higher frequencies above 10.156 Hz and 10 (24.4%) below 9.766 Hz. A typical shift to lower frequencies of the alpha spectral peak is shown in Figure 3. The alpha spectral peak had normal values in 8 patients without antiepileptic treatment and in 12 patients with treatment, these differences were not significant (*p* = 0.62).

## 4. Discussion

### 4.1. Clinical Characteristics

Our patients had a predominance of women over men, with a wide age range; the oldest patient was 68 years old, although the onset of the seizures was in his adolescence. A family history of epilepsy was present in only one-third of the patients. As reported in other articles, the average age of onset of the seizures was 13.6 years, during adolescence [22]. The presentation of the seizures followed the characteristic pathway started at 11 ± 1.5 years old with absence seizures, at 13.6 ± 1.71 years with myoclonic seizures, and at 15 ± 0.8 years with GTC seizures. Most of our patients had GTCs and myoclonic seizures, and very few patients had absences, which is a lower percentage than reported in other series [23,24,25,26].

JME seizures are characteristically present in the first one or two hours of the morning after awakening; our patients had their seizures at 7:39 a.m. on average; however, the awakening time is different amongst patients due to personal sleep habits. In addition, the time of awakening is closely linked to sleep start time [27]. Sleep deprivation, alcoholic beverage intake, and stress were the most frequent precipitant seizure factors; photic stimuli, menstruation, and playing videogames were rare causes. Sleep deprivation and alcohol intake on weekends were the two factors that, acting in a synergistic manner, most often caused seizures upon awakening [28].

Although JME was described 64-years ago [29] and is considered one of the most frequent epilepsies in young people, it is still not properly diagnosed. The above was already observed in previous studies, and ours is not the exception. Only 9.7% of patients had a JME diagnosis at the time of entering the study [30]. Similarly, only 26.8% of patients had treatment with valproate, which has been demonstrated to be the first-choice antiepileptic drug. The mean daily dose of valproate was 661.7 mg/day, which is consistent with the concept that JME patients require low doses of valproate to achieve adequate control of their epileptic seizures [31,32].

### 4.2. Paroxysmal Activity

The most frequent paroxysmal activity in our patients with JME was PSW complexes that were observed in all patients and fast SW complexes (73.1%), which have been consistently reported in previous studies. The analysis of the number of paroxysmal events and their duration has been less often reported. A duration of paroxysmal activity of 2 to 4 s was mentioned. In our study, a mean of 40.5 ± 62.6 paroxysmal events per hour and a mean duration of 1.7 ± 1.1s was found, but the duration was broad, between 0.3 and 15.2 s. The mean accumulated duration of the paroxysmal events was of 20.5 s, which represented 1.7% of all EEG recording. This data provides a clearer picture of paroxysmal activity in patients with JME which may be useful in electroencephalographic diagnosis of this kind of epilepsy. In previous studies, it has been observed that the paroxysmal activity of patients with JME can be asymmetric; less frequently, focal activity has been reported [33,34]. We found paroxysmal activity in symmetrical distribution in only 14.7% of patients. Asymmetrical paroxysmal activity was most frequent, shown in 85.3%, and right-side activity in the majority of patients (68.2%). Although this finding has been described previously, this is one of highest percentages that has been reported. Right-side asymmetric paroxysmal activity can be explained by some mechanism that is associated with working memory neuronal networks that have localization in right prefrontal dorsolateral regions; some working memory tasks were observed to precipitate seizures in JME patients [35].

### 4.3. Background EEG Activity

JME patients have long been considered to have normal EEG baseline activity. In our study, the background EEG activity was abnormal only in 31.7% patients upon visual EEG review. It could be hypothesized that the alterations in the background EEG activity may be secondary to the antiepileptic treatment, but the analysis of the differences between normal and abnormal EEGs in patients with and without antiepileptic treatment were not significant. There were two main alterations in EEG visual analysis that were found: generalized theta increases and alpha decreases were the most frequent abnormalities in these patients (46.1%). This pattern can be explained by alterations in brain maturation that characteristically delay the increase in alpha activity and the decrease in theta activity [36]. The other 46.1% of patients with abnormalities in visual EEG analysis had generalized beta increases. It can be hypothesized that an increase in beta activity is associated with stressful situations when a patient has new and shocking diseases. This is observed in generalized anxiety disorder, panic attacks, and other diseases [37].

However, QEEG analysis was used in some studies and was shown to increase the ability to detect abnormalities in background EEG activity [38]. In our group of patients with JME, the comparison between visual analysis and QEEG showed the low sensitivity and specificity of visual analysis to detect the subtle abnormalities in the background activity of the EEG. In addition, when neurometric analysis was used, more precision was added to finding subtle abnormalities [39,40]. The beta AP increase was the most frequent abnormality (53.5%), and theta (34.1%) and alpha (31.7%) increases were less frequent. In contrast, a decrease in the delta and theta bands (75.6% and 56.0%, respectively) were the most frequent abnormality. In the analysis of the proportion of patients without and with antiepileptic treatment who had normal or abnormal AP values of the delta, theta, alpha, and beta bands, only the differences in the alpha bands were significant. Therefore, it is assumed that antiepileptic treatment affects the results of the AP of the alpha frequency band and is not responsible for the modifications of the remaining frequency bands. The previous analysis allows us to establish that the increase in AP alpha affects approximately one third of patients with JME and another third with a decrease in AP alpha. The magnitude of these alterations, the brain regions most affected, and their possible causes will require future studies with an appropriate methodological design and different analysis tools. Furthermore, it is known that antiepileptic drugs affect the background EEG activity differently but the size of the sample of patients who were under treatment at the time of the EEG recording prevents a more in-depth analysis of which drugs are responsible for this effect [20].

The abnormalities in the MF of each frequency band were much less frequent. An increase in theta MF was observed only in 26.8% of patients, and a decrease in delta MF was found in 43.9%. In the spectral analysis, the alpha peak was found at 9.95 ± 0.85 Hz, with a greater amplitude in the occipital regions on the O1 and O2 electrodes. This spectral result may be explained because the majority of studies with EEG visual analysis obtained normal results since the alpha activity is the frequency that dominates the background of EEGs, so if this activity is normal in frequency, it is easy to conclude that all EEG activity is normal. On the other hand, the MF of the delta, theta, alpha, and beta bands had different but not significant results when the analysis of patients with and without antiepileptic treatment was performed. The same happened with the frequency of the spectral peak alpha. This does not mean that patients with JME do not have alterations in the background activity of the EEG, but rather that these alterations are not due to antiepileptic treatment and are most likely a consequence of the epilepsy itself [41].

## 5. Conclusions

We concluded that patients with JME started with absence seizures at 11.4 ± 1.5 years old, myoclonic seizures at 13.6 ± 1.71 years of age, and the GTC seizures at 15.1 ± 0.8 years. JME is a very common but little-known epilepsy with a three-year-delayed diagnosis. The seizures presented in the morning at 7:39 a.m. with sleep deprivation, alcoholic beverage intake, and stress states as the most frequent precipitating factors. The paroxysmal activity was of PSW and fast SW complexes with 40.5 ± 62.6 events per hour and a mean duration of 1.7 s. The asymmetric right-side paroxysmal activity in the frontal regions was present in 88.3% of patients. The visual analysis of the background EEG activity was abnormal in only 31.7% of patients and had sensitivity of 30.8% and specificity of 50.0%. With QEEG, the beta AP increase and delta AP decrease were the most frequent abnormalities that were found. But the increase or decrease in AP alpha that was present in one third of the patients with JME, respectively, was the only effect that was attributable to antiepileptic treatment. It is not responsible for the modifications of the remaining EEG frequency bands.

## Data Availability

Not Applicable.
